# Experimental study of the effects of the void located at the pile tip on the load capacity of rock-socketed piles

**DOI:** 10.1038/s41598-024-66831-2

**Published:** 2024-07-09

**Authors:** Xiaolin Zhao, Svetlana Melentijevic, Yupeng Shen, Zengkui Sun, Kaiyuan Wang, Jincui Xu, Zhiqiang Li

**Affiliations:** 1 School of Civil Engineering, Beijing Jiaotong University, Beijing, 100044 People’s Republic of China; 2 Facultad de Ciencias Geológicas, Universidad Complutense de Madrid, 28040 Madrid, Spain; 3 CCCC Highway Consultants Co., Ltd., Beijing, 100010 People’s Republic of China; 4 China Academy of Transportation Science, MOT of the PRC, Beijing, 100029 People’s Republic of China

**Keywords:** Rock-socketed pile, Void, Load capacity, Physical model test, Pile axial force, Unit pile side friction, Numerical simulation, Engineering, Civil engineering, Scaling laws, Computational methods

## Abstract

To address the design challenge of the rock-socketed piles posed by the void located below the pile tip, the physical laboratory model tests were designed and performed to simulate rock socketed piles using similar materials. The study investigates the behavior of the single pile under axial loading with the void located at varying distances from the pile tip. Through multi-level load tests, the variations of unit pile side friction, pile tip resistance, pile axial force and pile settlement are obtained for different positions of the void from the pile tip, as well as after grouting. Its comparison to the rock-socketed pile without void is performed as a reference to quantify the reduction in its bearing capacity. The results are presented in the form of graphs for different void positions and its grouting shows the influence on pile bearing capacity and emphasizes the importance of its detailed cautious investigation and introduction in the analysis. The 2D finite element modeling of the model pile-the void based on ABAQUS is performed to further investigate the influence of the void below pile tip on the bearing capacity of model pile, applying the Mohr Coulomb model as the constitutive model of rock mass behavior. The critical distance of the void below the pile tip is determined.

## Introduction

There are negative impacts of mining activities on civil engineering construction, with a significant concern being the safety threat posed by the collapse of the underground due to presence of voids^1^. Consequently, the heritage left by past mining activities has emerged as a growing concern, acknowledged worldwide as a matter deserving substantial attention^2^. The structural integrity and stability of the void is unknown, presenting the potential damages in various forms, and it is evident that these factors induce a major engineering and safety issue^3^.

The presence of voids reduces the load capacity of the strata above the voids, thus potentially causing damages to the infrastructures to be built, nevertheless, this doesn’t mean that constructing infrastructures above the void is unfeasible, depending on different requirements of different infrastructures regarding load capacity of the foundation to be built^4^. To prevent brittle and abrupt subsidence of the strata above the void, measures should be provided to address the situation. Common methods include^5–7^:

- A void is filled with concrete^8^, a technique aimed at supporting the strata above void and eliminating the brittle and abrupt subsidence.
- Employment of columns to locally support the strata above void, reducing the span of the void space and preventing the collapse of the strata above void^9^, such as grouted columns, masonry columns, large-diameter drilled columns, and piles.
- The surrounding rock of the void is strengthened through grouting materials, i.e. the discontinuities and fractures in the rock and soil of the fractured and bent zones of the strata above void are filled, forming a rock slab structure characterized by its high rigidity and integrity^1,10^, thus effectively resists the possible collapse of the void.
- Prior to utilizing the ground with presence of voids, proactive measures are taken to accelerate the activation of the void and induce subsidence in the strata above void. The strategy effectively eliminates underground voids that could influence ground safety.

Theoretically, the aforementioned methods can be employed for the remediation of the void beneath infrastructure. Nevertheless, due to technical and economic constraints, certain methods may prove difficulties for its implementation.

Rock-socketed piles (RSP) are common foundation, valued for their broad adaptability, strong load capacity, and ability to minimize uneven settlement^11^. Therefore, there is feasibility in constructing RSP above the void. The load capacity characteristics of RSP above the void differs from conventional RSP, primarily due to the following reasons: (i) The presence of void directly weakens the load capacity of the load-bearing layer at the pile tip, resulting in an increased settlement of the RSP. (ii) The existence of the void significantly diminishes the ultimate load capacity of RSP. (iii) Under the applied load at the pile top, the damage in the void is characterized by its brittle and abrupt feature. (iv) The damage to RSP in the void have significant consequences, and addressing such accidents entails considerable expenses. Hence, it is important to conduct research on the load capacity mechanisms, design theories, and calculation methods of RSP above the void. The research provides crucial guidance for the construction of such piles above the void.

Field static load tests are considered the most reliable tests for studying the load capacity mechanisms of piles, being extended to small scale laboratory load tests quite often to reproduce in situ conditions^12–16^. However, field tests are expensive, long-term, and have low repeatability, while physical model tests performed in laboratory have advantages such as^17,18^: (i) effectively protect the primary physical quantities of the test subject from external environmental interferences; (ii) effectively emphasize the primary conflicts within complex experimental procedures, facilitating the discovery of the fundamental traits of the model tests; (iii) requires easily manufactured equipment, convenient for its installation; (iv) can significantly save time and cost of full scale load tests considering difficulties that could be encountered in situ; (v) through appropriate method, model tests can explore certain performance aspects of actual engineering that remain unconstructed or are fundamentally challenging to study directly; (vi) can expedite the research process for phenomena in engineering with extremely gradual changes, and for rapidly changing phenomena, can also help prolong the research process; (vii) offer broad applicability, complementing other research methods to study similar issues, which aids in comparative conclusions, augmenting the scientific robustness of the study.

Therefore, many scholars choose the performance of small-scale laboratory tests as an alternative to full scale load tests. The behavior of piles regarding pile side friction (PSF) and pile tip capacity under different conditions of the sidewall roughness is studied in^16^. The influence of the pseudo-rock strength on the load distribution mechanism by friction on RSP is presented in^19^. The influence of the grouting on the contact of steel RSP on load-transfer mechanism is studied by experimental push-out tests and further parametric study by numerical models^20^. A model test for conducting bi-directional static load tests on piles, that enables the independent measurement of the load capacities of both pile shaft and pile base, eliminating the requirement for the support structures is presented in^21^. Model tests to explore the load capacity of RSP subjected to vertical loads, and additionally, based on the model test setup, the influence of the different socketing lengths (L/D ratios) and the pile-rock roughness profile on the load capacity of RSP is investigated in^22^. The load capacity and deformation behavior of inclined pile subjected to vertical and horizontal loads through model tests, using stainless-steel piles as model piles in a series of model tests is considered in^23^. The study of the shaft resistance, failure mechanism, load-settlement and load-transfer curves of steel pipe prebored and precast pile is performed in^24^. The uplift capacity of RSP under axial and oblique tension loading is studied by model experiment, its failure mode and load transfer mechanism in^25^. The influence of debris at the bottom of the RSP on the PSF distribution and load transfer mechanism is studied in^26^ by physical model experiments.

Many scholars have also studied the influence of different cave locations on the bearing performance of RSP through laboratory model tests and numerical simulations. Through static load test and finite element analysis, Chen et al. studied the influence of the height, roof span and roof thickness of the underlying karst cave on the vertical bearing characteristics of the bridge RSP^27^. Han et al. established a FEM model of karst cave-RSP under limestone stratum, and analyzed the bearing characteristics of RSP under the influence of pile length, pile diameter, limestone cohesion and roof thickness^28^. Through laboratory model test and numerical simulation Liang et al. analyzed the failure characteristics of RSP under horizontal load with presence of karst caves in front of pile and under pile^29^. Liang et al. studied the influence of various parameters of the cave on the bearing performance of the anti-slide pile by numerical simulation^30^. Wang et al. studied the factors affecting the bearing capacity of RSP in karst cave, analyzed the vertical and lateral bearing characteristics of RSP and the seismic dynamic characteristics of RSP under different distributions of karst caves, based on numerical simulations^31^. Considering the existence of the underlying karst cave, Yang et al. studied the pile foundation disturbance caused by foundation pit excavation by three-dimensional nonlinear finite element analysis^32^.

In order to deeply explore the impact of the void on the bearing capacity and the deformation characteristics of RSP, and propose engineering treatment measures, the study of RSP over the void, implemented for the construction of the Dujiashan grand bridge, spanning 2070.0 m, located in the northern Guizhou Province, was performed through the laboratory small scale test in this study. The bridge employs RSP with a diameter of 2.0 m, a length of 70.0 m and a designed load capacity of 44,000 kN. One of the piers of the bridge located above the void is studied. Based on the existing research results and in-situ geological data, it is assumed that the complex geometric shape of the void has a relatively small impact on the bearing capacity of RSP, thus, the planar geometric shape of the void being simplified to a semi-cylindrical shape. This study focuses on the impact of the void below pile tip on the bearing capacity of RSP. Through the application of physical model tests, the variations in pile axial force, unit PSF, pile tip resistance, and pile top settlement when the void is located at different distances from the pile tip is studied. It also analyzes the effectiveness by grouting of the void, offering a solid theoretical foundation for the design of similar engineering projects. Finally, a numerical model of the void located at the pile tip is established, and the correctness of the numerical model is verified by its comparison to the results of the laboratory model test. The critical void distance is determined by numerical model. The bearing mechanism of RSP when the void is located at different distances from the pile tip would be clarified in this study. By providing engineers with a more effective method for dealing with the void beneath RSP, the study aims to enhance the safety and reliability of engineering projects.

## Design of laboratory model tests

### Scaling factors and model materials

The prototype is modelled in the laboratory at Beijing Jiaotong University as a small-scale experimental test in the box of dimensions 1.0 × 1.0 × 1.5 m (length × width × height) (Fig. 2) to analyze the load transfer mechanism of piles constructed over voids. The behavior under axial loading of (a) the model pile without the presence of void, (b) the model pile with the presence of void at different distances from the pile tip and (c) the model pile over the grouted void, is studied and compared to each other. The position of the void is considered at 1, 2, 3, 4 and 5 pile diameters below the pile tip.

For the fulfillment of similarity between the prototype and laboratory model, the scaling factors^33,34^ of the small-scale model to the prototype is defined, such as the geometric scaling factor as *C*_*l*_ = 1/63, the bulk density scaling factor as *C*_*γ*_ = 1, the elastic modulus scaling factor as *C*_*E*_ = 1/63, etc. All relevant scaling factors of the model test are defined in Table 1.

**Table 1:** Scaling factors of the model test.

Type	Parameters	Dimension	Scaling factor
Material	Elastic modulus *E*	FL^−2^	63
	Poisson ratio *μ*	–	1
	Volumetric weight *γ*	FL^−3^	1
Geometry	Length *L*	L	63
	Rotation *θ*	–	1
	Strain *ε*	–	1
Force	Concentrated load *P*	F	63^3^
	Surface load *q*	FL^−2^	63
	Stress *σ*	FL^−2^	63

When conducting physical model tests involving rock material, natural rocks are rarely used because of the difficulties in obtaining consistent samples and the costs associated with preparing rock samples^35^. In order to accurately simulate the impact of the void located at the pile tip on the bearing performance of RSP, the model materials in the test are all prepared using similar materials. Several studies have been conducted to determine the optimal mixture of ingredients to produce the optimum material with engineering properties similar to natural rocks^36,37^. The stratum of the pile-rock physical model consists of an upper weak soil and a lower hard bedrock. The upper weak soil is a river sand characterized by main parameters, i.e. density and angle of internal friction. The lower hard bedrock is the bearing layer, and the main parameters used for its characterization are uniaxial compressive strength (UCS) and elastic modulus. The bedrock simulated by a mixture of river sand, gypsum, cement, light calcium carbonate, and water, with a mass ratio in the proportion of 1: 0.19: 0.06: 0.0575: 0.08. The artificial rocks have been used in physical model test and can achieve sufficient strength accuracy to simulate real rock. The grouting material used on site is cement-fly ash mortar, with a mass ratio of: *m*_cement_: *m*_fly ash_: *m*_water_: *m*_sodium silicate_ = 1:4:0.7:0.03. Its uniaxial compressive strength after 7 days of curing is 5.296 MPa. The grouting material used in the physical model test is configured from similar materials, with a mass ratio of: *m*_river sand_: *m*_gypsum_: *m*_cement_: *m*_water_ = 1:0.19:0.06:0.0575. Its uniaxial compressive strength after 7 days of curing is 0.089 MPa. Table 2 details relevant parameters of ground materials employed in the model.

**Table 2:** Properties of the prototype and model.

Material	Item	Prototype (concrete)	Model (high density polyethylene)
Pile	Diameter/mm	2 × 10^3^	32
	Length/mm	7 × 10^4^	1110
	Elastic modulus/MPa	3 × 10^4^	464
Material	Item	Prototype (soil)	Model (river sand)
Soil	Density/kN m^−3^	17.9	17.9
	Angle of internal friction/°	28	31
Material	Item	Prototype (bedrock)	Model (mixture of materials)
Moderately-weathered argillaceous siltstone	Density/kN m^−3^	26.5	26.1
	Elastic modulus/MPa	2626	40.341
	UCS/MPa	11.8	0.184
Material	Item	Prototype (cement-fly ash mortar)	Model (mixture of materials)
Cement-fly ash mortar	Density/kN m^−3^	21.0	20.0
	UCS/MPa	5.296	0.089

**Figure 1:**
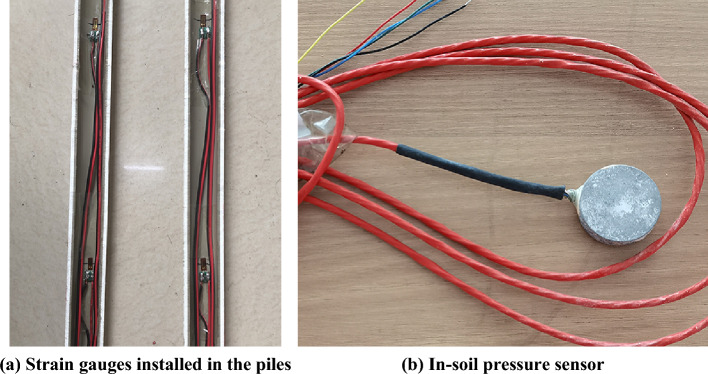
Strain gauges and in-soil pressure sensor.

**Figure 2:**
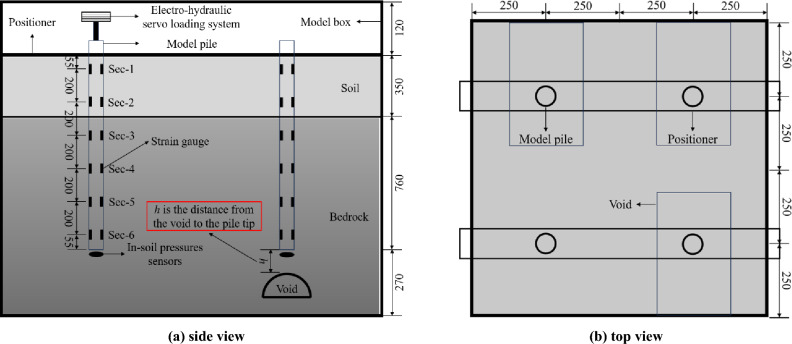
Schematic illustration of instrument layout and test device (unit: mm).

**Figure 3:**
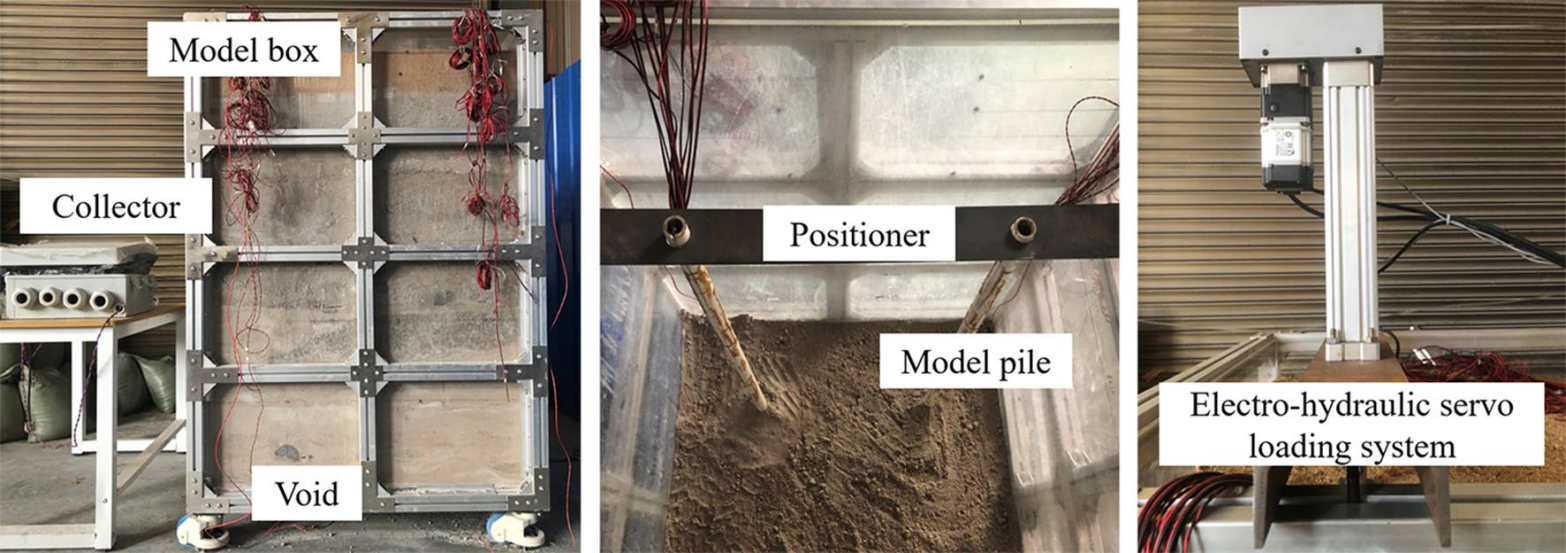
The model box facility of the RSP over void.

The single pile model is made of High Density Polyethylene tube, with an elastic modulus of 464 MPa^38^, a length of 1110 mm, an outer diameter of 32 mm, and a wall thickness of 5 mm. The model pile is split in half (Fig. 1a). Then, strain gauges are installed inside the pile at different depths along the pile length (Fig. 3a) so the pile axial force can be back-calculated through the measured strains. Afterwards, epoxy resin is used to bond the combined model pile and a nylon plug is pasted at the pile tip to seal the bottom. The contact of the pile and surrounding ground is simulated by gluing with a layer of fine sand. In order to facilitate the lead wires, a circular hole with a diameter of 6 mm is symmetrically opened at 30 mm below the pile top. Measuring the pile tip resistance of the model pile through the in-soil pressures sensor placed at the tip enabled the study of the load characteristics at the pile tip under compression. The in-soil pressure sensors (Fig. 1b) were calibrated at the beginning of the test. The positioning for installing the strain gauges and in-soil pressures sensor is illustrated in Fig. 2a.

It can be known that the vertical height range of the void is 0.7–3.1 m in the field survey data. The maximum height of the void at site is considered as 50 mm in the model test based on the geometric scaling factor as *C*_*l*_.

The axial force of the model pile at Section-i can be evaluated as

1 $$P_{\text{i}} = \sigma_{\text{i}} A = \varepsilon_{\text{i}} EA$$

where *σ*_i_ = pile axial stress at Section-i, *ε*_i_ = measured strain at Section-i, *E* = elastic modulus of model pile, *A* = cross-sectional area of model pile.

The PSF can be computed by

2 $$\tau_{\text{i}} = \frac{\Delta P}{{L_{\text{i}} d\pi }}$$

where *ΔP* = differential axial force of two sections, *L*_i_ = the length between two sections, and *d* = pile external diameter.

The pile-soil/rock relative displacement is expressed by

3 $$s_{\text{i}} = s_{\text{t}} - \sum\limits_{j = 1}^{i} {\frac{{L_{\text{i}} \left( {P_{\text{i}} + P_{{\text{i} + 1}} } \right)}}{2EA}}$$

where *s*_i_ = the pile-soil/rock relative displacement between two sections, *s*_t_ = the pile top settlement.

### Testing facility and instrumentation

**Figure 4:**
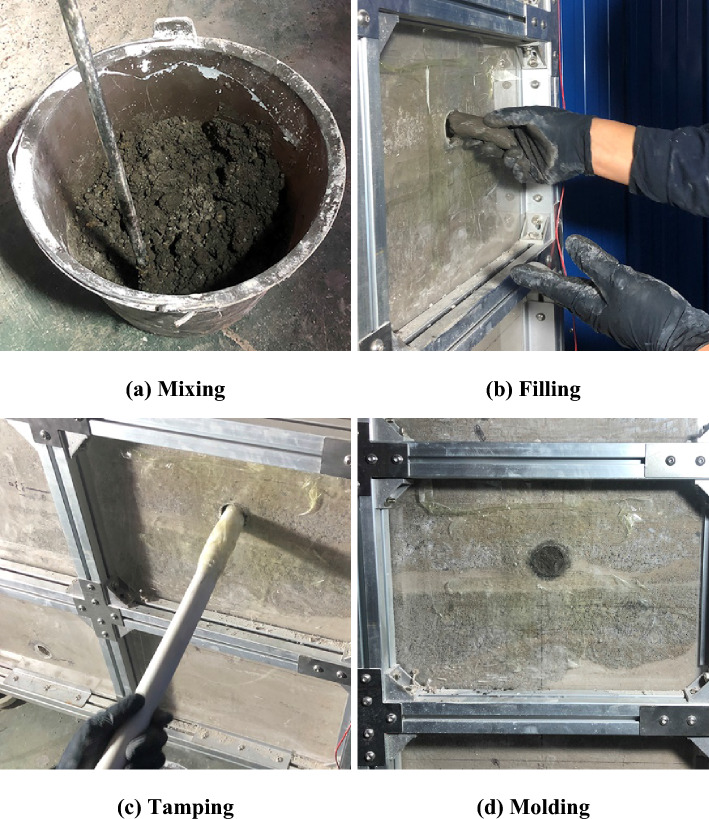
Grouting of the void.

Three series of experiments were performed in this study to evaluate the pile capacity: (a) no void in the ground, (b) the void at different distances from the pile tip, and (c) the void grouted with mortar. Figure 2 schematically shows the layout of model piles, being 11 the total number of small-scale piles studied. Figure 3 shows the pile model box facilities at the laboratory at Beijing Jiaotong University. The setup preparation of the experiment is as follows:

- The materials needed for simulating the bedrock of the prototype ground are prepared according to the specified proportions. The materials are mixed and stirred evenly after adding water, then the mixture is poured into the model box and compacted by a flat-bottom hammer and checked by a level ruler. The scale was labeled on the outer wall of the model box, compacting it every 100 mm of filling. When these materials reach the height of the void, the void mold is placed in its designated position, and filling and compaction of the material continue. After materials in the model box attains a specific strength, the void mold is withdrawn, creating a cavity.
- The in-soil pressure sensor is affixed onto the nylon plug at the pile tip to measure the pile tip resistance. The model piles are accurately placed in the designated position using a specially prepared positioner (a steel plate with a 35 mm diameter hole to secure the model piles). The materials of the bed rock are further filled into the model box and compacted. Four model piles are installed in the model box at distances of 500 mm in order not to affect each other.
- After completing the simulation of the void and the bedrock, materials simulating weak soil are introduced. River sand is evenly filled onto the bedrock and compacted every 100 mm.
- When the similar materials have been completely filled, the installation of the loading system commences. In this experiment, an electro-hydraulic servo loading system is utilized to apply axial loads (Fig. 3c). The slow maintained-load test method is applied to allow controlled staged-loading. The increment of each stage loading is 200 N. When the displacement of the model piles loaded at each stage are stabilized, the next stage of loading is carried out. When the displacement of a certain stage increases sharply, the loading is stopped.
- After completing the test of the RSP with no presence of the void and the tests of the RSP with different distances of the void from the pile tip, the pile bearing capacity test after grouting of the void is carried out. Remove the materials from the model box, refill it with new fillers, and the testing procedure described previously is repeated. After completing the filling with similar materials, the mortar is poured into the corresponding void location and compacted (Fig. 4).

## Experimental results of the model pile load test without and with presence of the void

### Pile top settlement

**Figure 5:**
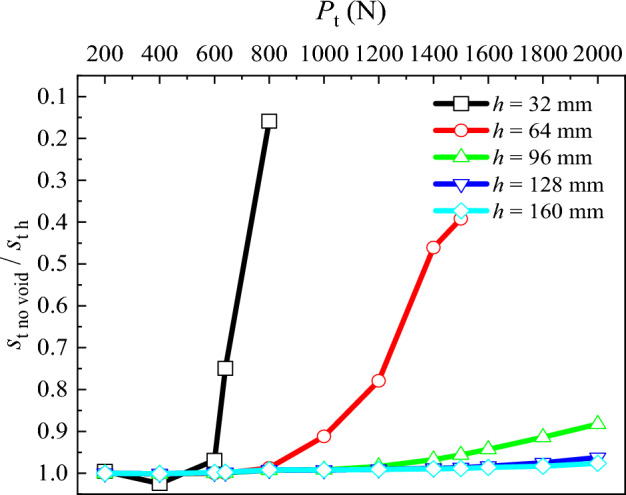
Comparative analysis of pile top settlement for different void positions.

To assess the influence of the presence of the void at different distances from the pile tip on the pile top settlement (*s*_t_), a graph illustrating the relation between the pile top settlement in absence and with presence of the void below pile tip at different distances (*s*_t no void_/*s*_t h_) under different loads applied at the pile top (*P*_t_) is plotted (Fig. 5). Upon comparison, it is evident that:

- For distances of void below pile tip of *h* = 160 and 128 mm, corresponding to distances of 5 to 4 pile diameters below the pile tip, with the increase in *P*_t_, there is no significant variation observed in the *s*_t no void_/*s*_t h_ for these two conditions, and the *s*_t no void_/*s*_t h_ remains close to 1.
- At *h* = 96 mm (3 pile diameters), the *s*_t no void_/*s*_t h_ decreases from 1.00 to 0.88 with increasing *P*_t_.
- Similarly, at *h* = 64 mm (2 pile diameters), the *s*_t no void_/*s*_t h_ decreases from 1.00 to 0.39 with the increasing load at pile top, reaching failure for the applied load of 1500 N.
- Additionally, at *h* = 32 mm (1 pile diameter), the *s*_t no void_/*s*_t h_ decreases from 1.00 to 0.16 with the increasing *P*_t_, without reaching the initially defined maximum load, being the failure observed at applied load of 800 N.

This indicates that with the decrease of *h*, *s*_t_ increases with the increasing *P*_t_. When the load bearing layer at the pile tip fails, significant settlement occurs, corresponding at this stage the pile top load to the maximum load capacity of the model pile. It can be concluded that with the decrease in the distance of the void to the pile tip (*h*), the maximum load capacity of the model pile diminishes.

### Pile axial force

**Figure 6:**
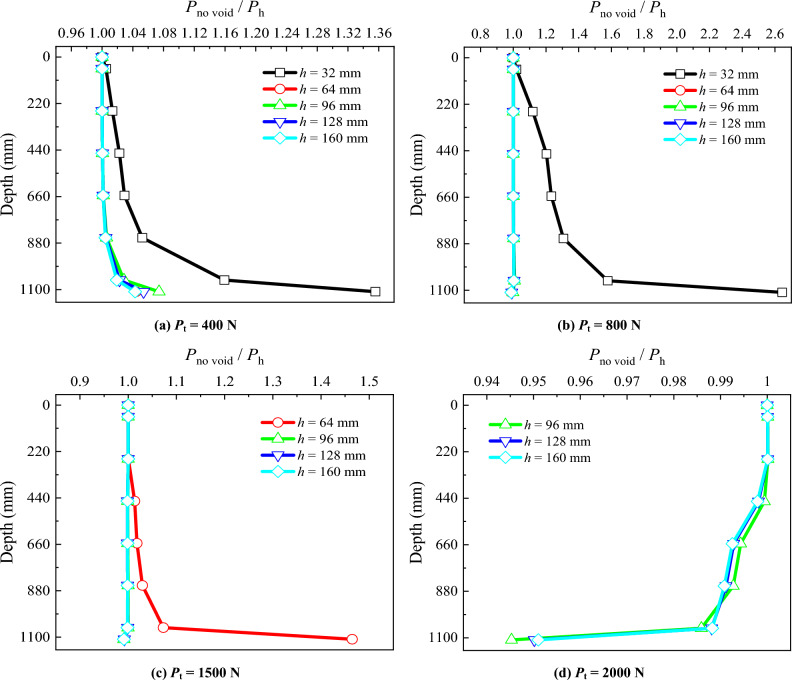
Comparative analysis of pile axial force for different void positions.

To study the impact of the position of the void at different distances from the pile tip on the pile axial force, a diagram depicting the *P*_no void_/*P*_h_ along the pile length is plotted for each applied load stage (Fig. 6). *P*_no void_ is the pile axial force in the absence of the void at the pile tip, and *P*_h_ is the pile axial force for the model pile with the presence of the void at different distances from the pile tip. Upon comparison, it is observed that:

- When *P*_t_ = 400 N, the *P*_no void_/*P*_h_ along the entire pile length increases from 1.00 to 1.35 as *h* = 32 mm, and the *P*_no void_/*P*_h_ for other positions of the void below the pile tip is similar.
- When *P*_t_ = 800 N and *h* = 32 mm, the load bearing layer at the pile tip fails, causing the increase of *P*_no void_/*P*_h_ from 1.00 to 2.64 along the entire pile length, while for other distances of the void to the pile tip, the *P*_no void_/*P*_h_ remains 1.00.
- When *P*_t_ = 1500 N and *h* = 64 mm, the load bearing layer at the pile tip fails, leading to the *P*_no void_/*P*_h_ increase from 1.00 to 1.45 along the entire pile length, while for other distances of the void to the pile tip, the *P*_no void_/*P*_h_ remains around 1.00. This suggests that under the pile top load, as *h* decreases, the *P*_no void_/*P*_h_ along the entire pile length increases. Consequently, the pile axial force decreases, posing a disadvantage to the continuous load capacity of the model pile.
- When *P*_t_ reaches the maximum load that could be applied by the loading system (2000 N), the load bearing layer with the void distance from the pile tip at *h* = 96, 128 and 160 mm (distances greater that 3 pile diameters) still provides resistance. Under these conditions, the *P*_no void_/*P*_h_ decreases from 1.0 to around 0.945 along the pile length.

### Pile tip resistance

**Figure 7:**
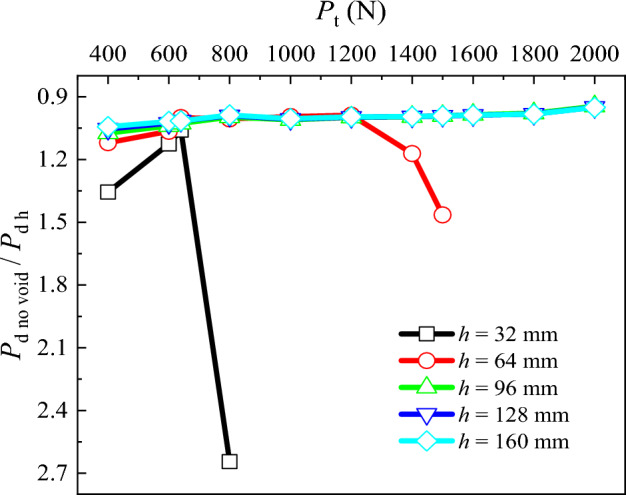
Comparative analysis of pile tip resistance for different void positions.

To analyze the effect of the presence of the void at varying distances from the pile tip on the pile tip resistance (*P*_d_), diagram illustrating *P*_d no void_/*P*_d h_ under different pile top loads is studied (Fig. 7). *P*_d no void_ is the pile tip resistance in the absence of the void at the pile tip, and *P*_d h_ is the pile tip resistance in the presence of the void at different distances from the pile tip. It can be observed that:

- In the case of distances corresponding to 5, 4 and 3 pile diameters, i. e. *h* = 160, 128 and 96 mm, with the increase in the pile top load, the relation *P*_d no void_/*P*_d h_ shows little variation, remaining close to 1.00.
- When *h* = 64 mm, with the increase in the pile top load, the *P*_d no void_/*P*_d h_ increases from 1.00 to 1.46, reaching failure at *P*_t_ = 1500 N.
- When *h* = 32 mm, the *P*_d no void_/*P*_d h_ decreases from 1.35 to 1.06 and then increases to 2.64 with the increase in the load at pile top reaching failure at *P*_t_ = 800 N.

As observed, and expected, with the decrease of *h*, the pile tip resistance diminishes with the increasing load at pile top.

### Unit PSF

**Figure 8:**
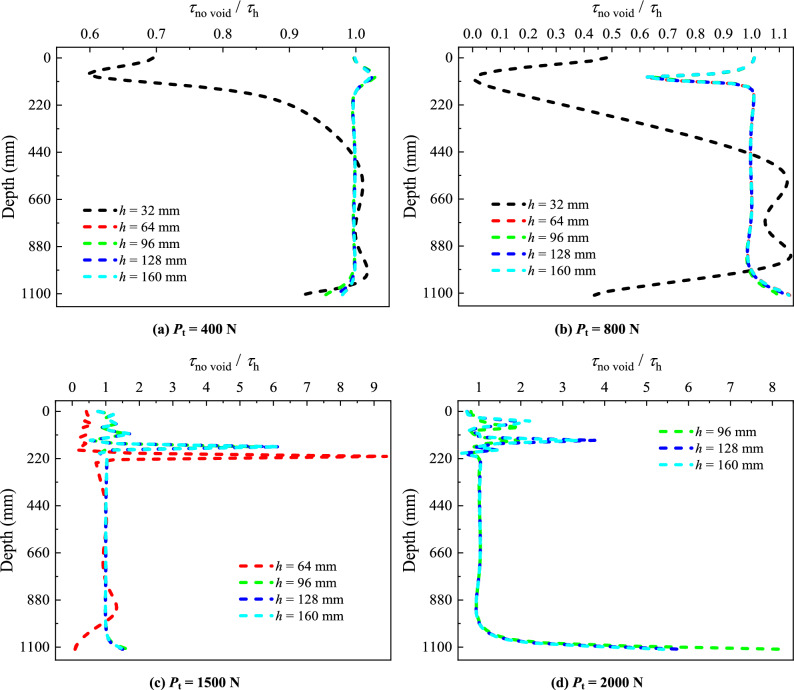
Comparative analysis of unit PSF for different void positions.

To investigate the influence of the void position at different distances from the pile tip on the unit PSF, diagrams illustrating *τ*_no void_/*τ*_h_ with depth are plotted (Fig. 8). *τ*_no void_ is the unit PSF in the absence of the void at pile tip, and *τ*_h_ is the unit PSF for the model pile with the presence of the void at different distances from the pile tip.

When *P*_t_ = 400 N (Fig. 8a), there are significant differences in *τ*_no void_/*τ*_h_ at *h* = 32 mm compared to other conditions. The *τ*_no void_/*τ*_h_ for other distances of the void to pile tip (*h* = 64, 96, 128 and 160 mm) remain close to 1.00 along the entire pile length, indicating no significant difference in unit PSF. The *τ*_no void_/*τ*_h_ of *h* = 32 mm exhibits noticeable fluctuations within the soil stratum (i. e. from the surface of the model pile down to depth of 350 mm) and remains below 1.00. This indicates that the unit PSF for *h* = 32 mm is higher than that of other positions within this range. In the range of rock stratum (depth from 350 to 1110 mm of the model pile), the *τ*_no void_/*τ*_h_ of *h* = 32 mm remains close to 1.00, similar to other positions.

When *P*_t_ = 800 N (Fig. 8b), failure occurs for the void position at *h* = 32 mm from the pile tip. Within the range of 0 to 500 mm of the model pile, the *τ*_no void_/*τ*_h_ of *h* = 32 mm is less than 1.00, indicating that the unit PSF for this condition is greater than that for other void positions. In the range of 500 ~ 900 mm of the model pile, the *τ*_no void_/*τ*_h_ is greater than 1.00, indicating a reduction in PSF within this range. The pile top load is primarily borne by the PSF down to depth of 500 mm. The pile top load transferred below 500 mm is minimal, so that the PSF near 1110 mm significantly decreases. For other conditions, the *τ*_no void_/*τ*_h_ shows significant fluctuation within the soil layer, approximately at the depth of 110 mm of the model pile. This suggests a sudden increase in unit PSF at this location and the soil stratum fails. Apart from this location, the *τ*_no void_/*τ*_h_ for other conditions remain close to 1.00 along the entire pile length, showing no significant variations.

When *P*_t_ = 1500 N (Fig. 8c), failure of the pile with void position at *h* = 64 mm from pile tip occurs. The relation *τ*_no void_/*τ*_h_ at *h* = 64 mm exhibits irregular fluctuation within the soil stratum, indicating soil stratum failure. Similar variations of the *τ*_no void_/*τ*_h_ within the soil stratum are observed for other void positions. Within the rock stratum, at *h* = 96, 128 and 160 mm, the *τ*_no void_/*τ*_h_ remains close to 1.00, indicating no influence of the void on pile unit side friction.

When *P*_t_ = 2000 N (Fig. 8d), the variations of *τ*_no void_/*τ*_h_ remain consistent for *h* = 96, 128 and 160 mm, all approaching 1.00 within the rock stratum. The pile top load is primarily borne by the unit PSF within the soil and rock stratum. The load transmitted to the pile tip is relatively small, resulting in lower PSF near the pile tip. Consequently, the *τ*_no void_/*τ*_h_ exceeds 1.00 in the proximity of the pile tip.

### PSF bearing ratio and pile tip resistance bearing ratio

The calculation formula for the PSF bearing ratio within the soil stratum (*ratio*_soil_) is presented by Eq. (4).

4 $$ratio_{{\text{soil}}} = \frac{{P_{\text{t}} - P_{h = 350mm} }}{{P_{\text{t}} }} \times 100\%$$

The calculation formula for the PSF bearing ratio within the rock stratum (*ratio*_rock_) is presented by Eq. (5).

5 $$ratio_{{\text{rock}}} = \frac{{P_{h = 350mm} - P_{\text{d}} }}{{P_{\text{t}} }} \times 100\%$$

The calculation formula for the pile tip resistance bearing ratio (*ratio*_tip_) is presented by Eq. (6).

6 $$ratio_{{\text{tip}}} = \frac{{P_{\text{d}} }}{{P_{\text{t}} }} \times 100\%$$

where, *P*_t_ is the load applied at pile top, *P*_h = 350 mm_ is the pile axial force at the boundary between the soil and rock strata, *P*_d_ is the pile tip resistance.

**Figure 9:**
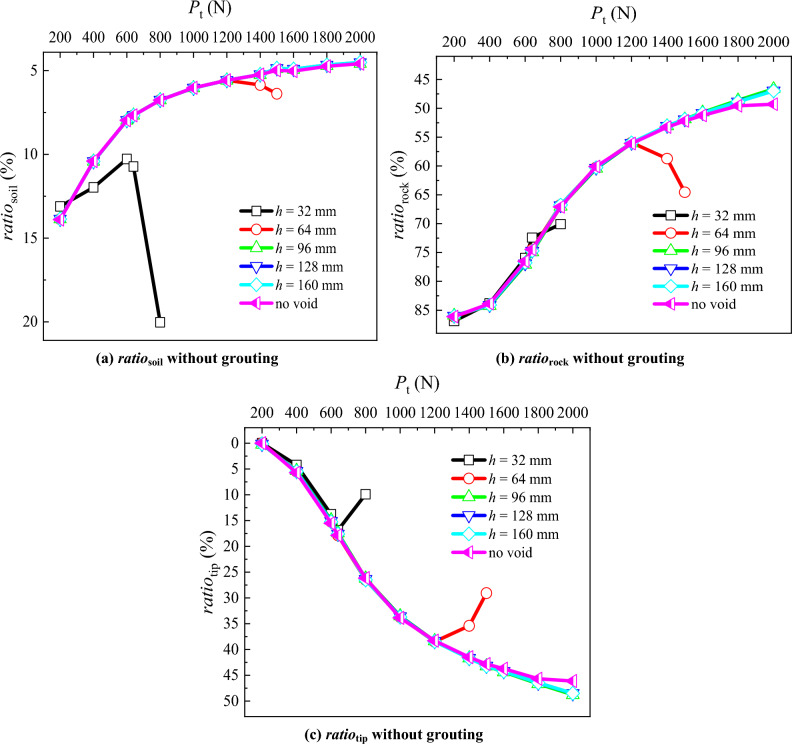
Comparative analysis of PSF bearing ratio and pile tip resistance bearing ratio for different void positions.

Comparing the situation of the RSP without the presence of the void, it can be observed that for void distances of *h* = 160, 128 and 96 mm, the *ratio*_soil_ (Fig. 9a) decreases from 13.87 to 4.55% with the increase of *P*_t_. When *h* = 64 mm, with the increasing *P*_t_, the *ratio*_soil_ decreases from 13.87 to 5.58%, and then increases to 6.38%. When *h* = 32 mm, with the increasing *P*_t_, the *ratio*_soil_ decreases from 13.11 to 10.73%, and then increases to 20.05%. This suggests that as *h* decreases, the *ratio*_soil_ decreases with the increasing *P*_t_. However, after the failure of the load bearing layer at the pile tip, the *ratio*_soil_ increases.

The *ratio*_rock_ is significantly higher than *ratio*_soil_ (Fig. 9b). With the increasing *P*_t_, the *ratio*_rock_ without the void decreases from 86.11 to 49.30%. Moreover, there is no significant difference in *ratio*_rock_ between the pile top loads of 1500 N and 2000 N. The *ratio*_rock_ at *h* = 160, 128 and 96 mm decreases from 85.97 to 46.86% with the increasing *P*_t_, which is lower than the *ratio*_rock_ without the void. When *h* = 64 mm, the *ratio*_rock_ decreases from 86.13 to 55.94% with the increasing *P*_t_, and then increases to 64.55%. When *h* = 32 mm, the *ratio*_rock_ decreases from 86.89 to 70.05% with the increasing *P*_t_.

With the increase of the *P*_t_, the *ratio*_tip_ increases (Fig. 9c), eventually reaching a level comparable to the *ratio*_rock_. In the absence of the void, the *ratio*_tip_ increases from 0 to 46.12% with the increasing *P*_t_. Additionally, there is no significant difference in the *ratio*_tip_ between the *P*_t_ of 1500 and 2000 N. At *h* = 160, 128 and 96 mm, the *ratio*_tip_ increases from 0.17 to 48.61% with the growing *P*_t_, surpassing the *ratio*_tip_ in the condition without the void at the pile tip. At *h* = 64 mm, the *ratio*_tip_ increases from 0 to 38.48% with the growing *P*_t_ and then decreases to 29.07%. Similarly, at *h* = 32 mm, the *ratio*_tip_ increases from 0 to 16.86% with the growing *P*_t_ and then decreases to 9.89%. As *h* decreases, the *ratio*_tip_ initially increases with the increasing *P*_t_. However, the *ratio*_tip_ decreases when the load bearing layer at the pile tip fails.

In summary, as *h* decreases, the pile top settlement increases, pile axial force decreases, and PSF increases. This indicates that the pile tip resistance provided by the load bearing layer gradually decreases. When the load transmitted to the pile tip exceeds the resistance that the load bearing layer can provide, the load bearing layer fails, resulting in significant settlement of the model pile, but the pile axial force is very small and the PSF is large. It is detrimental to the continued load capacity of the model pile. At this stage, the failure of the load transfer of the pile can be considered.

## Experimental results of the model pile load test after grouting the void

After completing the test of the RSP with no presence of the void and with different distances of the void from the pile tip, the materials in the model box were removed and refilled with new materials, and the testing procedure is repeated. After completing the filling with similar materials, the mortar is poured into the corresponding void location and compacted. After grouting, the weak soil, bedrock, and mortar are cured for a period of 7 days.

### Pile top settlement

**Figure 10:**
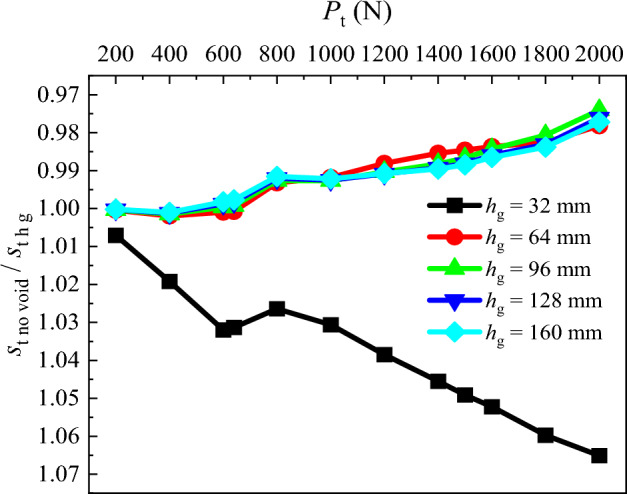
Comparative analysis of pile top settlement after grouting.

To investigate the variation of the pile top settlement if the grouting is performed to fill the void (*s*_t h g_) prior to the application of the pile top load (*P*_t_), the graph illustrating *s*_t no void_/*s*_t h g_ under different *P*_t_ is plotted (Fig. 10). *s*_t h g_ is the pile top settlement after grouting of the void situated at different distances from the pile tip. By comparison, it is evident that there are significant variations in pile top settlement after grouting of the void situated at different distances from the pile tip. At *h* = 32 mm, the *s*_t no void_/*s*_t h g_ is greater than 1.00, and it increases with the rise in *P*_t_. The *s*_t no void_/*s*_t h g_ for other distances of the void from the pile tip decrease from 1.00 as *P*_t_ increases. This indicates that after grouting, the pile top settlement at *h* = 32 mm is significantly smaller than that of other conditions.

### Pile axial force

**Figure 11:**
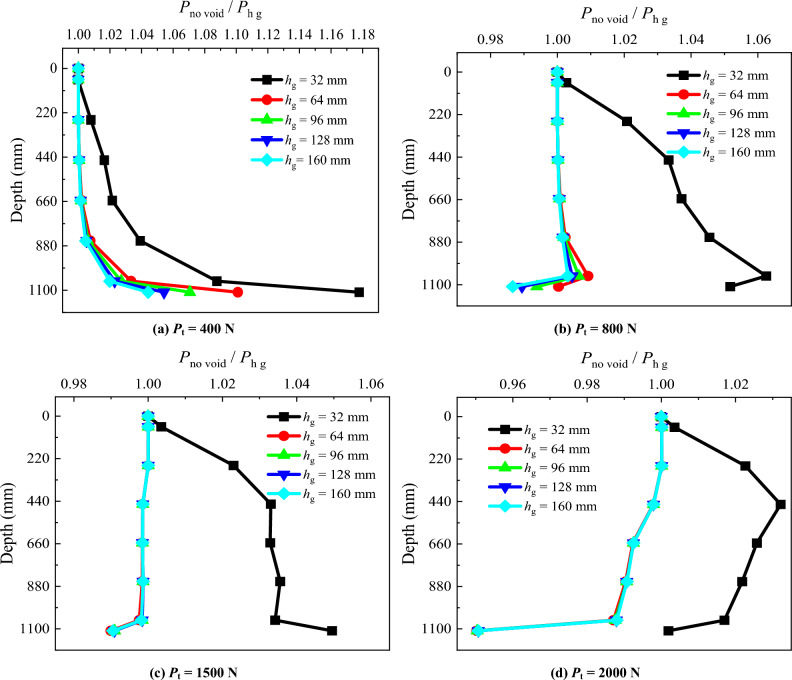
Comparative analysis of pile axial force after grouting.

In order to investigate the variation of the pile axial force after grouting, diagrams illustrating the pile axial force under various pile top loads after grouting were plotted (Fig. 11). *P*_no void_ is the pile axial force without void, and *P*_h g_ is the pile axial force after grouting. When *P*_t_ = 400 N, the *P*_no void_/*P*_h g_ of *h* = 32 mm increases from 1.00 to 1.18. The *P*_no void_/*P*_h g_ remains consistent along the pile length for other positions of the void distance from pile tip (*h* = 64, 96, 128, 160 mm). Near pile tip, as *h* decreases, the *P*_no void_/*P*_h g_ slightly increases for all conditions. With the increase in pile top load, the *P*_no void_/*P*_h g_ of *h* = 32 mm consistently exceeds 1.00 along the pile length. However, for *h* = 64, 96, 128 and 160 mm, the *P*_no void_/*P*_h g_ remains relatively stable along the pile length, approaching 1.00.

### Pile tip resistance

**Figure 12:**
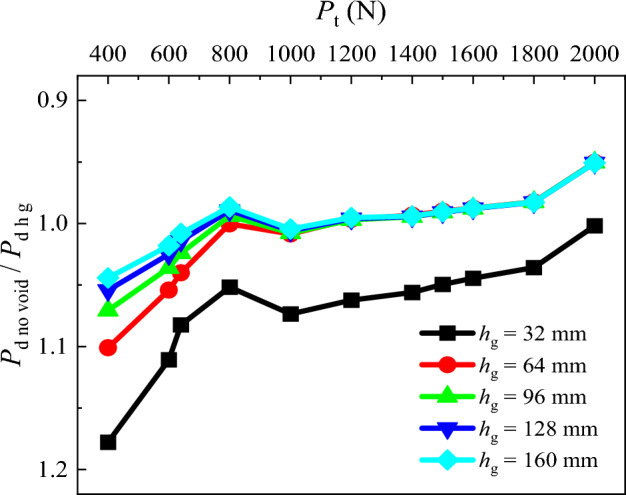
Comparative analysis of pile tip resistance after grouting.

To investigate the variation of the pile tip resistance after grouting, the graph illustrating *P*_d no void_/*P*_d h g_ under different pile top loads is plotted (Fig. 12). With the increase in the pile top load, the *P*_d no void_/*P*_d h g_ for all conditions follow a pattern of initial decreasing, and after reaching the load of 800 N remains constant. The *P*_d no void_/*P*_d h g_ of *h* = 32 mm is notably higher than other conditions.

### Unit PSF

**Figure 13:**
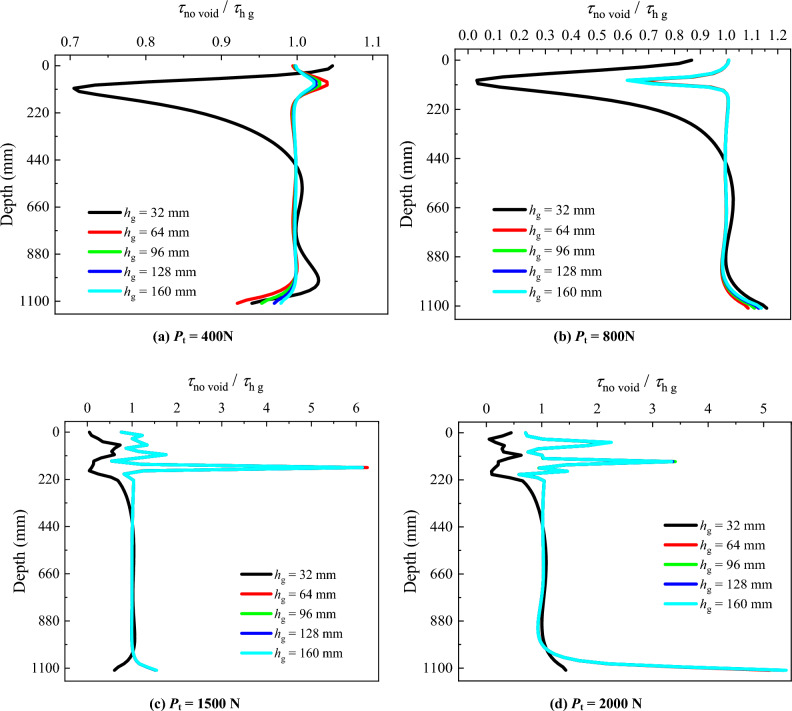
Comparative analysis of PSF after grouting.

To investigate the variation of unit PSF after grouting, the graph illustrating *τ*_no void_/*τ*_h g_ is plotted (Fig. 13), and the following can be concluded:

- When *P*_t_ = 400 N (Fig. 13a), the *τ*_no void_/*τ*_h g_ of *h* = 32 mm significantly decreases in the soil layer (at 110 mm of the model pile). For other conditions, the *τ*_no void_/*τ*_h g_ shows no significant variation along the pile length and remains close to 1.00.
- When *P*_t_ = 800 N (Fig. 13b), the *τ*_no void_/*τ*_h g_ decreases at 110 mm of the model pile for all conditions, being more significant for *h* = 32 mm. At other positions along the pile length, the *τ*_no void_/*τ*_h g_ remains close to 1.00.
- When *P*_t_ = 1500 and 2000 N (Fig. 13c,d), the *τ*_no void_/*τ*_h g_ of all conditions within the soil is intricate along the pile length, whereas the *τ*_no void_/*τ*_h g_ of all conditions within the rock remains consistently close to 1.00 along the pile length.

### PSF bearing ratio and pile tip resistance bearing ratio

**Figure 14:**
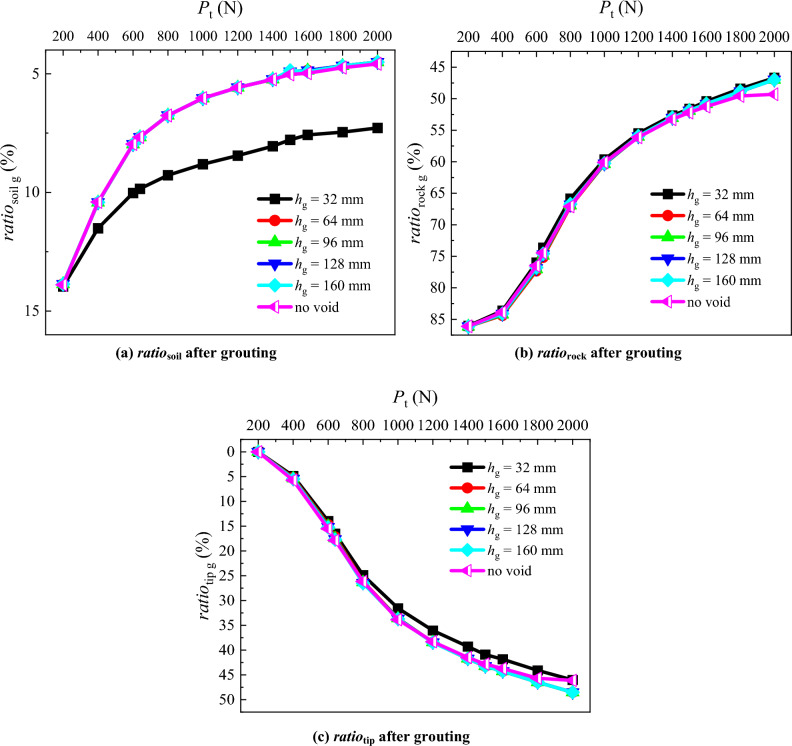
Comparative analysis of PSF bearing ratio and pile tip resistance bearing ratio after grouting.

In order to investigate the variation of the PSF bearing ratio and pile tip resistance bearing ratio if the grouting is performed to fill the void prior to the application of the pile top load, the graphs illustrating PSF bearing ratio and pile tip resistance bearing ratio under different *P*_t_ are plotted (Fig. 14). As the load applied at the pile top increases, the *ratio*_soil g_ after grouting for all conditions decreases. For *h* = 32 mm, the *ratio*_soil g_ after grouting decreases from 14 to 7.3%, while for other conditions, it decreases from 14 to 4.6%. Similar to the *ratio*_soil g_ after grouting, the *ratio*_rock g_ after grouting of *h* = 32, 64, 96, 128 and 160 mm decreases from 86.1 to 47% with the increasing pile top load. For the case without void, the *ratio*_rock g_ after grouting decreases from 86.1 to 49% as the pile top load increases. With the increasing pile top load, the *ratio*_tip g_ of *h* = 32 mm and the condition without void increases from 0 to 46.1%. Similarly, for *h* = 64, 96, 128 and 160 mm, the *ratio*_tip g_ increases from 0 to 48.6%.

In conclusion, after grouting, under the influence of the pile top loads, the settlement of the model piles reduces, and the pile tip resistances increases for all conditions. The effect is particularly significant for *h* = 32 mm, indicating a substantial enhancement in the load capacity of the load bearing layer at the pile tip.

## Numerical model

### Model information

**Figure 15:**
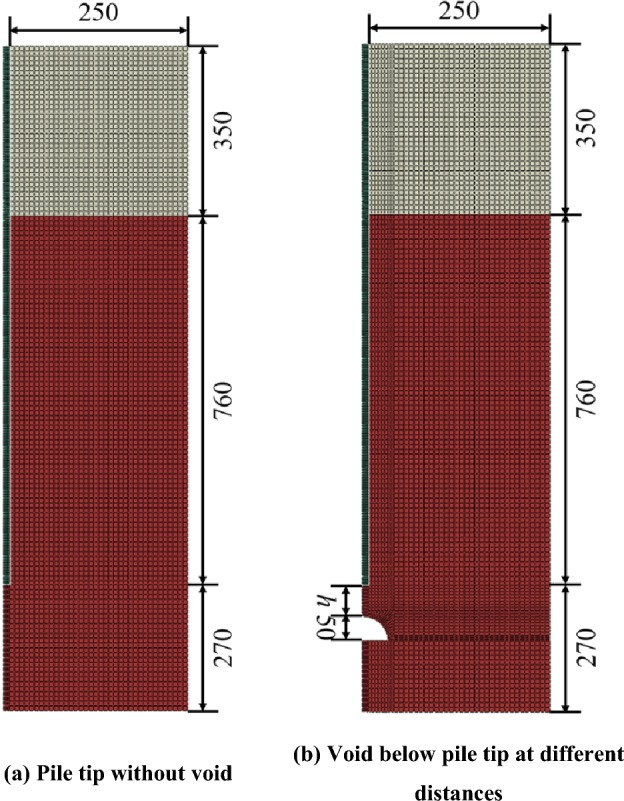
FEM model of pile without void and with void below pile tip at different distances (unit: mm).

In order to further investigate the influence of void at the pile tip on the bearing capacity of model pile, a symmetry FEM model is established in ABAQUS (Fig. 15). The position of the void considered is extended in comparison to laboratory test *h* = 0, 16, 32, 48, 64, 96, 104, 120, 126, 128 and 160 mm.

Pile and mortar are simulated using the linear elastic model. The Mohr–Coulomb model is used for describing the constitutive behavior of soil and rock. The mechanical parameters of the material in the numerical simulation are the same as those in the laboratory model test (Table 2). The friction coefficient for the pile-soil-rock interaction is 0.6. The top surface of model is free to displacement. The bottom of model is fixed in *X* and *Y* direction. The right surface of model is fixed in *X* direction. The left surface of model is a symmetry boundary condition and fixed in *X* direction. In order to simulate the real geostatic stress conditions, the geostatic stress is applied in the numerical model.

### Model validation

**Figure 16:**
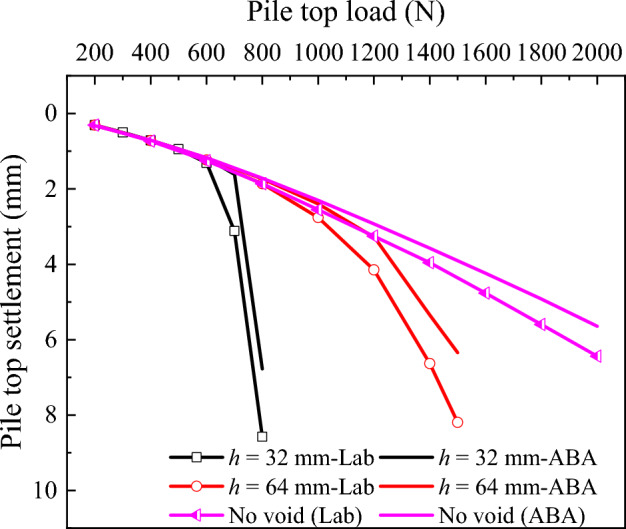
Pile top load-settlement.

**Figure 17:**
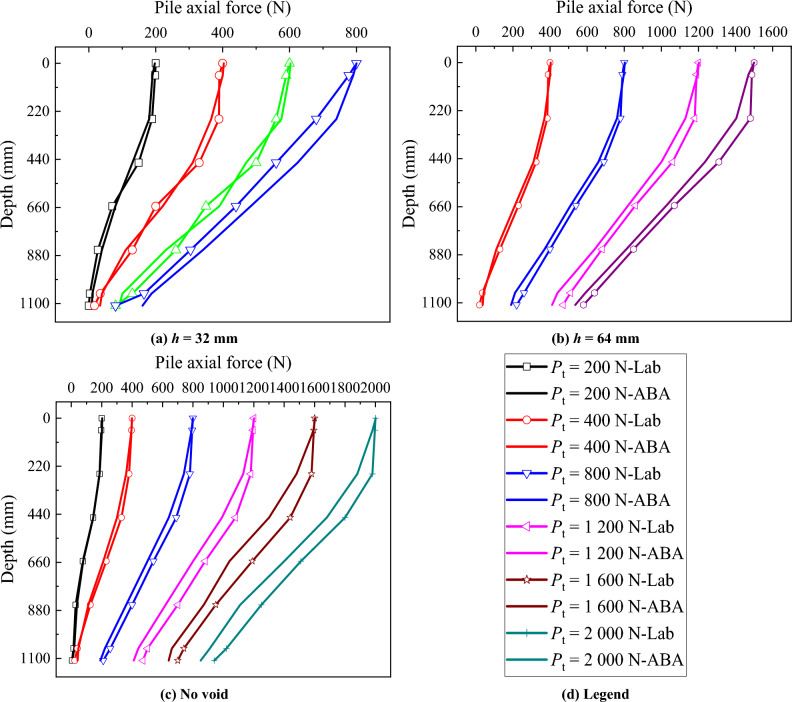
Pile axial force.

The pile top-load settlement, pile axial force and pile side friction, calculated by ABAQUS are compared with the results of laboratory model test (Figs. 16 and 17), for the case study with no void and with void at 1 and 2 pile diameter distances. It can be observed the close match of laboratory model tests, by that way the numerical model is used to extend the analysis of the influence of void at pile tip on the bearing capacity of piles.

### Ultimate bearing capacity and critical void distance

**Figure 18:**
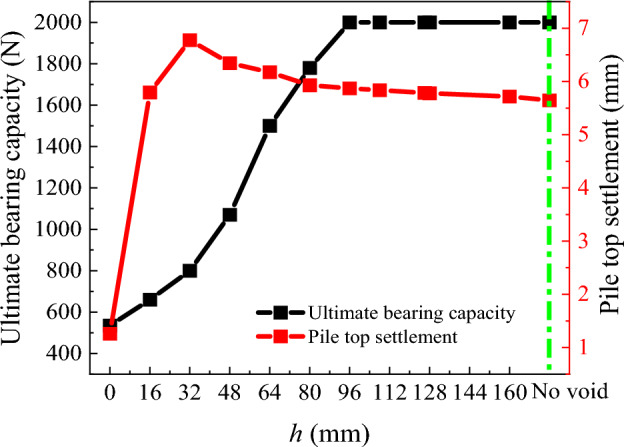
Ultimate bearing capacity and pile top settlement without void and with different void distance.

Considering the maximum pile top load as the ultimate bearing capacity of the model pile, the ultimate bearing capacity and the settlement of the model pile with no void and with varying distances of the void from the pile tip (*h*) are drawn (Fig. 18). As can be observed, with the increase of *h*, the ultimate bearing capacity of the model pile increases gradually. As *h* continues to increase, the pile top settlement gradually stabilized at about 5.7 mm under *P*_t_ = 2 000 N, thus *h* = 96 mm considered as the critical distance for the influence of the void below pile tip on bearing capacity.

**Figure 19:**
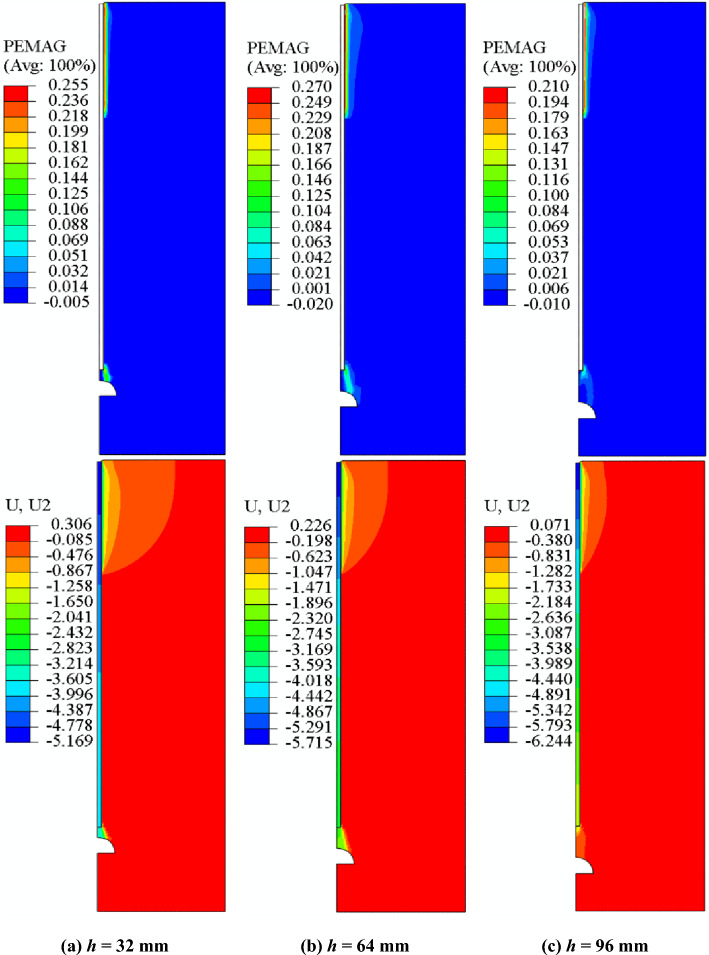
Plastic strain and vertical displacement.

Figure 19 shows the plastic strain and vertical displacement of the model at *h* = 32, 64, and 96 mm. Plastic strain results highlight the plastic area from the pile tip to the void, being a key area for analyzing the impact of the void on the bearing capacity of the model pile due to sudden failure of the pile when void is situated close to the pile tip. The vertical displacement of different *h* is similar, with larger displacement occurring in the soil and at pile tip. The vertical load on the pile top is large and the surrounding soil layer provides less PSF, resulting in a larger vertical displacement at the pile top. The significant vertical displacement at the pile tip is due to the presence of void, which weakens the bearing capacity of the bearing layer at the pile tip and increases the vertical displacement due to the lack of support conditions.

### The bearing ratio and ultimate bearing capacity reduction ratio

**Figure 20:**
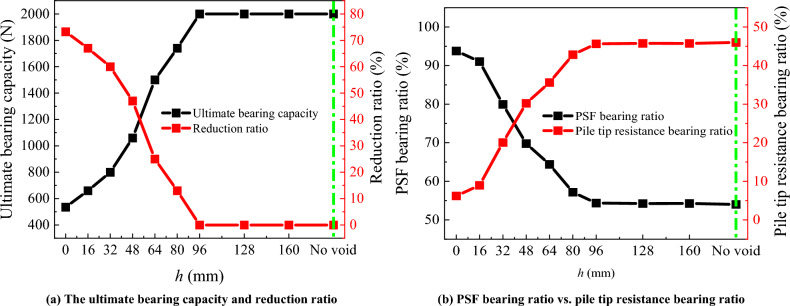
The ultimate bearing capacity and reduction ratio vs. bearing ratio.

The ultimate bearing capacity and reduction ratio for different void distances from pile tip are shown in Fig. 20. See Eq. (7) for the calculation of the reduction ratio.

7 $$\text{Reduction ratio} = \frac{{P_{{\text{No void-ultimate}}} - P_{{h\text{-ultimate}}} }}{{P_{{\text{No void-ultimate}}} }} \times 100\%$$

where, *P*_No void-ultimate_ is the ultimate bearing capacity of the model pile without void, and *P*_h-ultimate_ is the ultimate bearing capacity of the model pile with different distances from the void to the pile tip.

It can be seen from Fig. 20a, compared with the no void, the different distance between the void and pile tip will lead to the reduction of ultimate bearing capacity. With the increase of *h*, the ultimate bearing capacity increases gradually, and the reduction ratio decreases gradually. The main reason is that with the increase of *h*, the strength of pile tip bearing layer increases gradually.

In Fig. 20b, with the increase of *h*, the bearing ratio of PSF gradually decreases, while the bearing ratio of pile tip resistance gradually increases. Therefore, it can be judged that with the increase of *h*, the model pile changes from friction pile to end bearing pile. This means that when the mechanical properties of the pile tip bearing layer are consistent, the larger the *h* is, the bearing capacity of the pile bottom bearing layer will gradually increase, and a higher pile tip resistance can be raised.

## Conclusion

The focus of this study is the influence of the void at the pile tip on the vertical bearing capacity of RSP. This reduction of the load bearing capacity is studied by the analysis of the model pile laboratory tests, using the prototype of RSP over void in the Dujiashan grand bridge engineering project. The experimental setup, design methodology, material preparation, testing procedures and analysis of results for the presence of the void at different distances from the pile tip are described. The influence of the presence of the void at different distances from the pile tip on the settlement, pile axial force, unit PSF and pile tip resistance of the model piles under multiple levels of loading at pile top is studied. Also, its comparison to the case when the void is grouted is performed.

The burial depth and spatial structure of voids are complex. The idealization of void is uncertain and extrapolations from physical model testing to practical recommendations for real field conditions is not an easy task. Given the scaling dimensions, installation of pile and location of the void, extrapolating the results from a given “*h*” in mm (which is not a comparative ratio) in laboratory scale to real practical field conditions is not direct and thus, the overall conclusions of the paper are only applicable to the tested conditions.

The main conclusions are as follows:

1. Under the same load applied at pile top, with the reducing distance between the void and the pile tip, the settlement of the pile top and the unit PSF increase, and the pile axial force and the pile tip resistance decrease. When the load bearing layer at the pile tip is damaged, the PSF bearing ratio suddenly increase, and the pile tip resistance bearing ratio suddenly decrease. When *h* = 32 m the *ratio*_tip_ decreases by 41.35%, and for *h* = 64 mm the *ratio*_tip_ decreases by 17.92%.
2. After grouting the void, the settlement of the pile top and the unit PSF decrease, while the pile axial force and pile tip resistance increase. With the increase of load applied at pile top, the PSF bearing ratio gradually decrease, and the pile tip resistance bearing ratio gradually increase.
3. As expected, the presence of the void leads to pile top settlement of the model pile, which is caused by the presence of the void at the pile tip results in a decrease in the load capacity of load bearing layer at the pile tip. This effect is more significant when the distance of the void from the pile tip is less than 2 pile diameters, with the failure of the load bearing layer at pile tip for the void distance of 1 pile diameter from the load bearing layer at pile tip and *P*_t_ = 800 N, while for the distance of 2 pile diameters and *P*_t_ = 1500 N, the load bearing layer at the pile tip fails.
4. The presence of the void leads to an increase in the PSF bearing ratio and a decrease in the pile tip resistance bearing ratio of the model pile, which indicates that the presence of the void leads to an increase in the PSF, and a decrease in the pile axial force. The load applied at the pile top is carried by the PSF, so the bearing capacity of the model pile is not fully utilized.
5. After grouting, the load capacity of the load bearing layer at the pile tip significantly increases, and the *ratio*_tip_ also increases significantly, enhancing the load capacity of the model pile. This indicates that grouting can enhance the bearing capacity of the load bearing layer at the pile tip, so that the load capacity of the model pile can be fully utilized. Therefore, grouting proves to be an effective method in enhancing the load capacity of the pile with the void below the pile tip.
6. When the mechanical properties of the pile tip bearing layer are consistent, *h* increases, the ultimate bearing capacity of the model pile increases significantly, at the same time, the pile tip resistance bearing ratio increases, the PSF bearing ratio decreases, and the model pile changes from friction pile to end-bearing pile. Therefore, careful consideration should be given to the length of the pile to maintain a safe distance of the void to the pile tip. The critical void distance obtained in this paper is 96 mm.

In the practical engineering, the scope, burial depth, and spatial structure of the void are intricately complex. Under such intricate geological conditions, RSP can result in diverse mechanical responses. In light of this, this study only considers one specific condition amid these complexities: the presence of the void at different distances from the pile tip, which is not comprehensive in previously published literature. The results of this study can elucidate the mechanical response of RSP when the void is located at different distances from the pile tip. However, these findings are not applicable for describing the mechanical response of RSP under other conditions. In order to investigate the mechanical response of RSP under different void conditions, it is imperative to conduct thorough research on the mechanical response of RSP under different void conditions, such as the void at the side of RSP and RSP passing through the void.

## Data Availability

The datasets generated and analysed during the current study are available from the corresponding author on reasonable request.
